# RNA-binding proteins and translational regulation in axons and growth cones

**DOI:** 10.3389/fnins.2013.00081

**Published:** 2013-05-23

**Authors:** Hanna Hörnberg, Christine Holt

**Affiliations:** Department of Physiology Development and Neuroscience, University of CambridgeCambridge, UK

**Keywords:** RNA-binding proteins, local translation, axon outgrowth, axon guidance, synapse formation

## Abstract

RNA localization and regulation play an important role in the developing and adult nervous system. In navigating axons, extrinsic cues can elicit rapid local protein synthesis that mediates directional or morphological responses. The mRNA repertoire in axons is large and dynamically changing, yet studies suggest that only a subset of these mRNAs are translated after cue stimulation, suggesting the need for a high level of translational regulation. Here, we review the role of RNA-binding proteins (RBPs) as local regulators of translation in developing axons. We focus on their role in growth, guidance, and synapse formation, and discuss the mechanisms by which they regulate translation in axons.

## Introduction

Spatial localization of mRNA is a well conserved mechanism for restricting gene expression to a specific subcellular site in many cell types across animal and plant phyla (Condeelis and Singer, 2005; Holt and Bullock, 2009). In neurons, localization and translational regulation of mRNA plays a key function in dendrites and post-synaptic compartments (Bramham and Wells, 2007), and mounting evidence points to a similarly important role in axons (Jung et al., 2012). The response to several guidance cues require local protein synthesis in the tip of the growing axon, the growth cone (GC), (Campbell and Holt, 2001; Wu et al., 2005; Leung et al., 2006; Piper et al., 2006; Yao et al., 2006) and axonal mRNA translation is critical for axon survival (Hillefors et al., 2007; Cox et al., 2008; Yoon et al., 2012) and regeneration (Zheng et al., 2001; Verma et al., 2005). A large number of mRNAs are found in both growing (Andreassi et al., 2010; Zivraj et al., 2010) and mature axons (Taylor et al., 2009; Gumy et al., 2011), with some transcripts restricted to specific neuronal subtypes, axonal compartments (Zivraj et al., 2010), or developmental time points (Zivraj et al., 2010; Gumy et al., 2011).

Different guidance cues ultimately lead to the translation of distinct subsets of mRNAs (Wu et al., 2005; Leung et al., 2006; Piper et al., 2006; Yao et al., 2006), yet, puzzlingly, cause an increase in the activity of markers of global translation in the GC (Campbell and Holt, 2001; Leung et al., 2006; Piper et al., 2006). This begs the question of how translation of specific mRNAs is locally regulated. The specificity is likely mediated, at least in part, via RNA-binding proteins (RBPs). RBPs comprise a large family of proteins that form ribonucleoprotein (RNP) complexes with their target mRNAs and can act as cytoskeletal adaptors and/or translational silencers to transport their cargo to subcellular locations (Besse and Ephrussi, 2008). Once on site, RBPs can either act as translational repressors or activators of their mRNA targets, thus providing a way to control translation spatially and temporally. Here, we review the role of RBPs as regulators of local protein synthesis in the axon during development, from axon elongation, to axon guidance and synapse formation in target-arrived axons. Lastly, we discuss the possible mechanism by which RBPs regulate the specificity of local translation in axons and GCs.

## Axonal growth cone RBPs revealed by proteomic analysis

RBPs are widely expressed in the central nervous system (CNS) and many exhibit region-specific expression in the developing brain, suggesting that RBPs may play a major role in establishing cell-type specific function during development (McKee et al., 2005). However, most of our knowledge of the function of RBPs in neurons stems from distinct cellular or dendritic compartments, and although RBPs have been found in axons (Zhang et al., 2001; Rossoll et al., 2002, 2003; Leung et al., 2006; Price et al., 2006; Yao et al., 2006; Christie et al., 2009; Akten et al., 2011), their full repertoire has not been determined and little is known about their abundance and distribution in axonal compartments.

An unbiased proteomic study has been performed recently on GCs from whole rat embryonic brain (Estrada-Bernal et al., 2012) and we have interrogated this dataset to determine the repertoire of RBPs. Interestingly, our analysis indicates that about 1% of all GC proteins are putative RBPs. This estimate is likely to be an under-representation because the experimental design of the study favors proteins expressed in the majority of GCs without taking into account any regional-or cell-specific expression of RBPs. Consistent with this line of reasoning, RBPs previously identified in axons, such as HuD (Akten et al., 2011; Fallini et al., 2011), the Fragile X mental retardation protein (FMRP) (Antar et al., 2006; Price et al., 2006; Christie et al., 2009; Akins et al., 2012) and cytoplasmic polyadenylation element-binding protein (CPEB) (Kundel et al., 2009) were not identified in this screen, indicating that these RBPs may only be present in specific subsets of axons. Nonetheless, the study provides unparalleled insights into the repertoire of GC RBPs. Out of the 22 putative RBPs identified, only two, zipcode binding protein 1 (ZBP1, also known as IMP-1 and Vg1RBP) and survival motor neuron 1 (SMN) have previously been identified in GCs (Zhang et al., 2001, 2003, 2006; Leung et al., 2006; Fallini et al., 2011; Welshhans and Bassell, 2011). The single largest group of RBPs, comprising about 50% of all RBPs identified in the GCs, were the heterogenous nuclear ribonucleoprotein family (hnRNP) family of RBPs, a large family of RBPs that varies greatly in both function and structure (Han et al., 2010). To date, only one family member has previously been identified in axons and GCs (Rossoll et al., 2002, 2003; Glinka et al., 2010), but their striking enrichment in the GC proteome suggests that they may have a widespread role in developing axons. Members of the hnRNP family have also been identified in post-synaptic densities (Jordan et al., 2004; Zhang et al., 2012), indicating that they may serve key functions in both pre- and post-synaptic compartments. However, it is worth nothing that the hnRNPs enriched in post-synaptic densities differs from the hnRNPs most abundant in GCs (Zhang et al., 2012).

Of the other RBPs identified in GCs, four were RNA-recognition motif (RRM) containing proteins previously identified for their role in splicing and transcription (Imai et al., 1993; Patturajan et al., 1998; Kataoka et al., 2000; Guo et al., 2003; Cazalla et al., 2005; Chuang et al., 2011; Albers et al., 2012). Many hnRNPs also have known nuclear functions, and it is interesting to note that the majority of RBPs identified in this study, including hnRNP K (Expert-Bezancon et al., 2002; Lynch et al., 2005; Stains et al., 2005), U (Kukalev et al., 2005; Huelga et al., 2012), F (Min et al., 1995; Martinez-Contreras et al., 2006; Huelga et al., 2012), E1 (Kim et al., 2005; Akker et al., 2007), H1 SMN (Pellizzoni et al., 2002) and RNA binding motif protein 8a (RBM8a, also known as Y14) (Kataoka et al., 2000; Chuang et al., 2011; Albers et al., 2012), have well-established nuclear functions as regulators of splicing and transcription. In fact, of all RBPs found in GCs, only ZBP1 is best known for its cytoplasmic function. This raises the intriguing possibility that many neuronal RBPs may have a dual role both in the nucleus and cytosol. Interestingly, both proteins and mRNAs of splicing factors have been found in GCs (Zivraj et al., 2010; Estrada-Bernal et al., 2012), suggesting that axonal mRNA regulation may be more complex than previously thought.

## RBP-mediated local regulation of axon growth, guidance, and synapse formation

While the role of RBPs in dendrites and post-synaptic compartments has traditionally received more attention (Bramham and Wells, 2007; Swanger and Bassell, 2011), several studies are starting to focus on the role of RBPs in axons. Some of these RBPs, like hnRNP R and SMN, appear to localize mainly in axons outside of the nucleus. Others, such as FMRP and ZBP1, have both dendritic and axonal functions. In this section, we review the role RBPs play as local regulators during axon growth, guidance, and synapse formation (Table 1).

**Table 1:** **RBPs in axons**.

**RBP**	**Species**	**Cell type**	**Function**	**Target mRNA**	**References**
CPEBs	Rat	Hippocampal neurons	Axon growth, branching	*β-catenin*	Kundel et al., 2009
	*X.laevis*	RGCs	Axon guidance		Lin et al., 2009
FMRP	Mouse	Hippocampal neurons	GC motility	*map1b*	Antar et al., 2006
			Axon guidance		Li et al., 2009
			Synapse formation		Hanson and Madison, 2007
	*Drosophila*	Mushroom body motor neurons	Branching, synapse formation	*futsch*	Pan et al., 2004; Tessier and Broadie, 2008
			Branching, synapse formation		Zhang et al., 2001; Gatto and Broadie, 2008
FMRP,	Rat	Developing brain	Synapse formation?		Christie et al., 2009; Akins et al., 2012
FXR2,					
FXR1					
hnRNP	Mouse	Motor neurons	Axon growth	β-*actin*	Rossoll et al., 2003; Glinka et al., 2010
R	Zebrafish		Axon growth, synapse formation		Glinka et al., 2010
HuD	Mouse	Motor neurons	Axon growth, branching	*cpg15*	Fallini et al., 2011
SMN	Mouse	Motor neurons	Axon growth	β-*actin*	Rossoll et al., 2003
		Motor neurons	Branching, synapse formation		Kariya et al., 2008; Kong et al., 2009
		RGCs			Liu et al., 2011
	Zebrafish	Motor neurons	Axon growth,		McWhorter et al., 2003
	*X.tropicalis*	Motor neurons	Branching		Ymlahi-Ouazzani et al., 2010
TDP-43	Mouse	Motor neurons	Axon outgrowth	*futsch*	Fallini et al., 2012
	*Drosophila*		Synapse formation		Godena et al., 2011; Lin et al., 2011
	Zebrafish		Axon growth, branching, synapse formation		Kabashi et al., 2010
ZBP1	*X.laevis*, chick, mice	RGCs, cortical neurons	Axon guidance	β-*actin*	Zhang et al., 2001; Leung et al., 2006; Yao et al., 2006; Sasaki et al., 2010

### Axon growth

Axon outgrowth and the continuous regulation of axon growth are key steps during axon guidance and regeneration. Two RBPs associated with neurodegenerative disorders affecting motor neurons have been implicated as local regulators of axon growth suggesting that translational regulation in axons during this process may be broadly crucial for the survival and health of motor neurons. These RBPs are SMN and TDP-43.

SMN is a ubiquitously expressed RBP most known for its role in assembling small nuclear ribonucleoprotein (snRNP) complexes involved in splicing (Burghes and Beattie, 2009). Depletion of SMN is the cause of spinal muscular atrophy (SMA) and loss of SMN leads to degeneration of motor neurons. However, why the loss of a ubiquitously expressed gene causes a specific defect in motor neurons is not well understood. SMN has been detected in axons (Rossoll et al., 2002; Zhang et al., 2003, 2006; Fallini et al., 2011), and cultured motor neurons from a SMN mouse model display axonal defects including reduced axon growth, smaller GCs and reduced levels of β-*actin* mRNA in the axon and GC (Rossoll et al., 2003). In zebrafish and *Xenopus tropicalis*, knockdown of SMN leads to truncated motor neuron development *in vivo* (McWhorter et al., 2003; Ymlahi-Ouazzani et al., 2010). SMN can interact with several other RBPs (Mourelatos et al., 2001; Rossoll et al., 2002; Wang et al., 2002; Piazzon et al., 2008), and is thought to regulate translation indirectly via these interactions as SMN itself lacks any known RNA-binding domains. One of these RBPs, hnRNP R, is reduced in GCs and axons of cultured motor neurons lacking SMN (Rossoll et al., 2003), and depletion of hnRNP R in zebrafish gives a similar phenotype to SMN knockdown (Glinka et al., 2010). hnRNP R can associate with β-*actin* 3′ UTR and co-localizes with β-*actin* in GCs (Glinka et al., 2010). Knockdown of hnRNP R leads to a decrease in β-*actin* mRNA levels in GCs but no change in total mRNA levels, suggesting that hnRNP R specifically alters the subcellular location of β-*actin* mRNA. Overall, these findings suggest that SMN and hnRNP R co-regulate β-*actin* mRNA localization and translation in the distal axon during axon growth in motor neurons.

The TAR DNA binding protein 43, TDP-43, is also implicated in axonal regulation of motor neuron outgrowth. TDP-43 is mostly a nuclear DNA/RNA-binding protein involved in many parts of mRNA post-transcriptional regulation such as splicing, stability, and transport (Lee et al., 2012). TDP-43 is implicated in neurodegenerative diseases such as amyotrophic lateral sclerosis (ALS) and frontotemporal lobar degeneration (FTLD-U) where TDP-43 is found in large insoluble granules in the cytoplasm, but the pathogenesis of these granules is not clear. Apart from its nuclear location, TDP-43 has been found in axons of motor neurons where it co-localizes with other RBPs (Fallini et al., 2012). Axonal TDP-43 levels increase after BDNF stimulation in cultured motor neurons, and depletion of TDP-43 increases axon length and branching (Fallini et al., 2012). However, in mouse neuroblastoma neuro-2a cells TDP-43 depletion inhibits neurite outgrowth (Iguchi et al., 2009), and in zebrafish embryos zTDP-43 depletion causes reduced axon length in motor neurons (Kabashi et al., 2010), suggesting that TDP-43 may have different roles during neuronal development in different neuronal populations and species.

### Axon guidance

**Figure 1:**
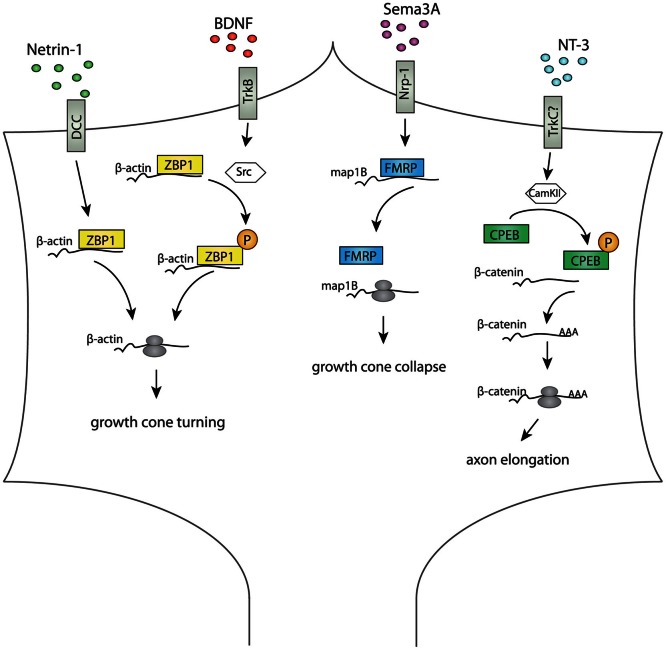
**Diagram summarizing RBP-mediated regulation of different cue-stimulated responses in axonal growth cones.** In the growth cone, RBPs mediate translation of specific mRNAs after cue stimulation. Netrin-1 induces ZBP1 localization and translation of β-actin close to the source of Netrin-1, and this is crucial for growth cone turning (Leung et al., 2006; Lin and Holt, 2007). BDNF induces Src-mediated ZBP1 phosphorylation, β-actin translation and growth cone turning toward the BDNF source (Yao et al., 2006; Sasaki et al., 2010). Growth cone collapses and Map1B mRNA translation in response to Sema3a is attenuated in axons depleted of FMRP, suggesting a role for FMRP in Sema3A-mediated axon guidance (Li et al., 2009). NT3 induces CamKII mediated phosphorylation of CPEB1, which activates polyadenylation and translation of β-catenin mRNA crucial for axon elongation and branching (Kundel et al., 2009).

Local protein synthesis plays a key role during axon guidance *in vitro* (Campbell and Holt, 2001; Wu et al., 2005; Leung et al., 2006; Piper et al., 2006; Yao et al., 2006) and *in vivo* (Leung et al., 2013) and although many mRNAs have been identified in axons (Taylor et al., 2009; Andreassi et al., 2010; Zivraj et al., 2010; Gumy et al., 2011), the identity of those that are actively translated and how they are regulated is less clear. RBPs are needed to transport mRNA to the GCs, but what regulatory role they play in the GCs during axon guidance is not well known. Some RBPs, depicted in Figure 1, can mediate the response to guidance cues by regulating local translation of their target mRNAs. ZBP1 was the first RBP found to regulate axon guidance, and its local regulation of β-*actin* mRNA in response to guidance cues is conserved in several species (Zhang et al., 2001; Leung et al., 2006; Welshhans and Bassell, 2011). In *Xenopus laevis*, the ZBP1 ortholog, Vg1RBP, mediates turning toward the attractive guidance cue Netrin-1 (Leung et al., 2006) and to brain-derived neurotrophic factor, BDNF (Yao et al., 2006). Stimulation of retinal ganglion cell (RGC) axonal GCs by a Netrin-1 gradient induces polarized movement of Vg1RBP toward the Netrin-1 source, and this is accompanied by an asymmetrical increase in activated eIF-4E-binding protein 1 (4EBP1) and β-*actin* translation (Leung et al., 2006). A BDNF gradient also leads to asymmetric β-*actin* and Vg1RBP localization in spinal cord neuron GCs, and preventing the β-*actin*-ZBP1 interaction abolishes both Ca^2+^-mediated attraction and repulsion (Yao et al., 2006). This suggests that ZBP1 is crucial for regulating both the translation and spatial location of β-actin during GC turning. Together these two studies gave the first insight into how an RBP can spatially restrict translation in the GC. Translational dysregulation of β-actin can cause morphological defects in axons of several types of neurons (Zhang et al., 2001; Huttelmaier et al., 2005; Leung et al., 2006; Yao et al., 2006; Welshhans and Bassell, 2011), and several axonal RBPs have β-actin mRNA among their targets (Zhang et al., 2001; Rossoll et al., 2003; Huttelmaier et al., 2005; Leung et al., 2006; Glinka et al., 2010; Welshhans and Bassell, 2011), suggesting that translational regulation of β-actin may be of particular importance in axons. In dendrites, ZBP1-mediated dysregulation of β-actin perturbs branch development (Perycz et al., 2011), but whether or not ZBP1 has a similar function in axonal branching is not known.

FMRP is best known for its role as a translational regulator in the post-synaptic compartment, but it is also gaining attention for its role in axons (Christie et al., 2009; Deng et al., 2011; Till et al., 2011; Akins et al., 2012). FMRP is present in axons and GCs (Antar et al., 2006), and knockdown of FMRP in hippocampal neurons leads to reduced GC motility, excess filopodia (Antar et al., 2006), and attenuated collapse in response to the repulsive guidance cue Semaphorin 3A (Sema3A) (Li et al., 2009). Sema3A stimulation increases the levels of phosphorylated eukaryotic translation initiation factor eIF4E and MAP1b translation in distal axons, but this increase is abolished in FMRP deficient neurons (Li et al., 2009), suggesting a role for FMRP in axons during Sema3A-mediated GC steering via regulation of MAP1b translation in the GC.

### Axon arborization and synapse formation

Translational regulation is crucial for synaptic function, and a number of cognitive disorders are linked to mRNA dysregulation. For example, Fragile X syndrome (FXS), the most common form of inherited mental retardation, is caused by the loss of FMRP function and subsequent dysregulation of its target mRNAs (Bassell and Warren, 2008). Although FXS is thought to be caused mainly by the loss of FMRP function in the post-synaptic compartment, several lines of evidence suggest that FMRP may also have a pre-synaptic role at the synapse. FMRP binds to many mRNAs encoding pre-synaptic proteins (Akins et al., 2009; Darnell et al., 2011), and several pre-synaptic proteins are differentially regulated in fmr1 knockout (KO) mice (Klemmer et al., 2011). In *Drosophila*, the mushroom body neurons of dFMRP null mutants have over-branched axonal arbors and form abnormal synapses, (Pan et al., 2004; Tessier and Broadie, 2008), and abnormal pre-synaptic structures at the neuromuscular junction (NMJ) (Zhang et al., 2001; Gatto and Broadie, 2008). Furthermore, in a mosaic mouse model of FXS, neurons lacking FMRP function form fewer synaptic connections than wild type neurons, suggesting that pre-synaptic FMRP function may determine the likelihood of forming a synapse (Hanson and Madison, 2007). Pre-synaptic expression of FMRP appears restricted to a subset of neuronal circuits where it is present in granules (Fragile X granules; FXGs) in association with its paralogs FXR2p and FXR1p (Christie et al., 2009; Akins et al., 2012). The expression of these granules peak during the time of synapse formation and pruning (Christie et al., 2009; Akins et al., 2012), indicating a possible pre-synaptic role for FMRP and its paralogs during synapse formation in a subset of axon population.

Disruption of SMN also causes pre-synaptic abnormalities. In a mouse model of SMA, axons at the NMJ terminals are poorly arborized and display abnormal neurofilament accumulation in the nerve terminals (Kariya et al., 2008). Furthermore, SMN knockdown causes abnormal synaptic transmission (Kariya et al., 2008; Kong et al., 2009), lower synaptic vesicle density at the pre-synaptic site (Kong et al., 2009) and a reduction of Ca_v_2.2 Ca^2+^ channels at the NMJ (Jablonka et al., 2007). Interestingly, knockdown of hnRNP R leads to a similar phenotype (Glinka et al., 2010), indicating that SMN and hnRNP R may co-regulate translation both during axon growth and synapse formation. SMNs' function has mostly been studied in motor neurons, but similar defects have been reported in the retina of a mouse SMA model (Liu et al., 2011), suggesting that perhaps SMN has a conserved role in axon elongation and connectivity in several axon populations. SMN may regulate synapse formation partially via co-regulation of the candidate plasticity-related gene 15 (cpg15), an activity-regulated protein with key functions during branching and synaptogenesis in the NMJ. SMN can interact with HuD (Akten et al., 2011; Fallini et al., 2011), an RBP known to bind to and regulate cpg15 expression (Wang et al., 2011), and both SMN and HuD co-localize with cpg15 in axons. Disruption of SMN function reduces the amount of *cpg15* mRNA, and overexpression of cpg15 partially rescues the SMA phenotype in a zebrafish model (Akten et al., 2011). Together these studies suggest a crucial role for SMN and HuD mediated *cpg15* mRNA regulation in axons during synapse formation at the NMJ.

TDP-43 has also been shown to cause defects in axonal branching and synapse formation at the NMJ. Depletion of TDP-43 causes an increase in synaptic boutons at the NMJ in *Drosophila* (Lin et al., 2011), and immature and excessive branching in zebrafish (Kabashi et al., 2010). In *Drosophila*, the defects are associated with a decrease in the microtubule stabilizing protein futsch, a MAP1B ortholog. dTDP-43 can interact directly with *futsch* mRNA, and dTDP-43s RNA-binding property is essential for its function in synapse formation (Godena et al., 2011). Furthermore, dTDP-43 depletion decreases futsch protein in distal boutons, but *futsch* mRNA levels was unchanged, suggesting a role for dTDP-43 in translational regulation of futsch (Godena et al., 2011).

## Translational regulation by RBPs in the axon and growth cone

How do RBPs repress translation in the GC, and how is translation activated? Translational repressors are found in RNPs (Kim-Ha et al., 1995; Nakamura et al., 2004; Paquin et al., 2007) and when bound to their targets these repressors can regulate translation by either blocking translation elongation, or, most often, translational initiation. ZBP1 can block translation initiation by inhibiting recruitment of the 60S subunit (Huttelmaier et al., 2005), FMRP is thought to block translation elongation by recruiting the eIF4E-binding protein CYFIP1 (Napoli et al., 2008) and the post-synaptic RBP, Pumilio, regulates the abundance of eIF4E at the NMJ (Menon et al., 2004).

RBPs may also regulate translation via modulating the length of the poly(A) tail of mRNA. CPEB controls translation by polyadenylation and directly binds the CPE sequence in the 3′UTR of its target mRNAs (Richter, 2007). Blocking polyadenylation attenuates the collapse response to Sema3A in *Xenopus* retinal axons (Lin et al., 2009), and blocking CPEB1's function in hippocampal neurons causes a reduction in NT3-induced β-actin translation in the GC, possible via Ca^2+^ mediated inositol triphosphate (IP3) and Ca^2+^/calmodulin-dependent protein kinsase II (CamKII) activation (Kundel et al., 2009). This suggests that regulation of poly(A) tail length may be a common way for guidance cues to regulate translation of specific mRNAs.

Stimulation of GCs with protein synthesis-inducing guidance cues, such as Netrin-1 and Sema3A, leads to the activation of global translation, as indicated by 4EBP1 and mTOR activation, yet they each stimulate the translation of a distinct set of mRNAs (Wu et al., 2005; Leung et al., 2006). Furthermore, guidance cues can stimulate translation globally while repressing specific transcripts (Yoon et al., 2012), and both translation reporters and newly synthesized protein can be localized to specific compartments in the GC (Leung et al., 2006; Yao et al., 2006). How translational specificity is achieved and how it is spatially localized in the GC is largely unknown.

Signal-mediated phosphorylation of RBPss present in the GC may provide a way to regulate translation of specific mRNAs. BDNF induces Src-mediated phosphorylation of ZBP1, and blocking this step attenuates local β-actin translation and GC turning (Sasaki et al., 2010). Src is activated asymmetrically toward the BDNF source (Yao et al., 2006), indicating that localized activation of distinct set of RBPs may provide both spatial and temporal control over translation. FMRP activity is also regulated by phosphorylation (Narayanan et al., 2008; Muddashetty et al., 2011; Coffee et al., 2012), suggesting that phosphorylation could be a common mechanism for releasing RBP-mediated repression upon cue stimulation.

RBPs can also mediate translational regulation via small non-coding RNAs such as microRNAs (miRNAs). miRNAs can associate with RBPs (Schratt et al., 2006; Edbauer et al., 2010), and RBPs are known to regulate the abundance of miRNAs (Michlewski et al., 2008; Xu et al., 2008, 2011; Xu and Hecht, 2011). In dendrites, there is evidence that miRNAs can act locally as translational repressors (Schratt et al., 2006). miRNAs and RNA-induced silencing complex (RISC) components have been found to associate with FMRP (Caudy et al., 2002; Jin et al., 2004; Muddashetty et al., 2011), and FMRP may depend on miRNAs to repress some of its targets (Muddashetty et al., 2011). HuR can interfere with miRNA-mediated repression in cell culture, both as an antagonist of miRNA repression (Bhattacharyya et al., 2006) and in a cooperative manner to help facilitate repression (Kim et al., 2009). Interaction studies suggest that HuR may regulate the efficiency of several miRNAs (Mukherjee et al., 2011), and it would be interesting to see if its neuronal family member, HuD, can act in a similar fashion. miRNAs have been found in the distal axon (Natera-Naranjo et al., 2010; Han et al., 2011; Dajas-Bailador et al., 2012), and seem to play a role in guidance cue responses. Knockdown of Dicer leads to axon guidance defects in the visual system in mice (Pinter and Hindges, 2010), and knockdown of the miRNA miR-124 leads to guidance defects of RGC axons caused by an attenuated response to Sema3A (Baudet et al., 2012). However, miRNA-RBP mediated regulation is a relatively novel concept and whether or not RBPs can regulate miRNA repression in axons and GCs is not yet known.

Another intriguing possibility is that receptor-ribosome interactions may be used to restrict translation spatially and confer additional translation specificity. The Netrin-1 receptor, DCC, can interact directly with the translational machinery by forming complexes with ribosomal subunits. This interaction is disassociated upon Netrin-1 stimulation to promote DCC-mediated translation (Tcherkezian et al., 2010), suggesting a possible mechanism to spatially restrict cue-induced translation to a specific subcellular compartment.

## Future perspectives

RBPs are beginning to emerge as important players in the pre-synaptic compartment during the building of neuronal circuits, but many questions still remain. The list of axonal RPBs is still incomplete, and little is known of their mRNA targets in axons. New techniques such as crosslinking immunoprecipitation (CLIP) (Ule et al., 2005) and high throughput sequencing-CLIP (HITS-CLIP) (Licatalosi et al., 2008) will be valuable in future studies for identifying RBP-mRNA complexes in different axon populations and developmental time points. Moreover, further studies on the interactions of RBPs with other post-transcriptional regulatory pathways are needed to help gain insight into how translational specificity is achieved in the GC. For example, it will be important to investigate if guidance receptor coupling to the translational machinery (Tcherkezian et al., 2010) is a common way of restricting translation locally, and to understand the interplay between other pathways such as miRNAs, RBP phosphorylation and mRNA polyadenylation in regulating the spatiotemporal control of local protein synthesis in response to extrinsic cues.

The observation that many axonal RBPs are best known for their nuclear roles suggests that some axonal RBPs may have dual functions in the nucleus and cytosol. Nuclear mRNA processing is important for subsequent cytosolic localization (Giorgi and Moore, 2007), and the axonal and nuclear localization of some RBPs may provide a platform to coordinate pre-mRNA processing and cytosolic translational regulation (Bava et al., 2013). The presence of splice-regulating RBPs in axons suggests the intriguing possibility that some pre-mRNA processing may occur locally in axons. Indeed, cytoplasmic splicing has been identified in neurons (Bell et al., 2010), and splice components localized to dendrites retain their ability to splice RNA (Bell et al., 2010). Splice factors have been found in GCs (Estrada-Bernal et al., 2012), but whether they are involved in splicing or other processes is not known.

Finally, although it is increasingly clear that local translation occurs in navigating axons and post-synaptic compartments, its role in target-arrived axons is much less understood. Transcripts of synapse-associated proteins are commonly present in axons (Zivraj et al., 2010), and pre-synaptic translation has been implicated in synapse development (Taylor et al., 2013), synaptic plasticity (Yin et al., 2006; Deng et al., 2011; Je et al., 2011; Johnstone and Raymond, 2011; Till et al., 2011) and arborization (Dajas-Bailador et al., 2012; Donnelly et al., 2013). Translational dysregulation is thought to underlie several neurodevelopmental and neurodegenerative disorders (Bear et al., 2004; Liu-Yesucevitz et al., 2011; Santini et al., 2013; Taylor et al., 2013). RBPs such as FMRP, SMN and TDP-43 have all been linked to neurological diseases, and their presence in axons suggests that axonal translation may play a role in disease pathology. Elucidating how presynaptic translation influences synapse formation and the role RBPs play in this process will further deepen our understanding of how neuronal circuits are formed and maintained in the developing brain.

### Conflict of interest statement

The authors declare that the research was conducted in the absence of any commercial or financial relationships that could be construed as a potential conflict of interest.
